# Fecal microbiota transplantation in inflammatory bowel disease patients: A systematic review and meta-analysis

**DOI:** 10.1371/journal.pone.0238910

**Published:** 2020-09-18

**Authors:** Luciane de Fátima Caldeira, Helena H. Borba, Fernanda S. Tonin, Astrid Wiens, Fernando Fernandez-Llimos, Roberto Pontarolo

**Affiliations:** 1 Pharmaceutical Sciences Postgraduate Research Program, Universidade Federal do Paraná, Curitiba, Brazil; 2 Laboratory of Pharmacology, Department of Drug Sciences, Faculty of Pharmacy, University of Porto, Porto, Portugal; University of Tennessee Health Science Center, UNITED STATES

## Abstract

**Objectives:**

Current evidence on fecal microbiota transplantation for inflammatory bowel disease is inconclusive. We conducted a systematic review to gather evidence on the efficacy and safety of fecal microbiota transplantation for inflammatory bowel disease.

**Methods:**

Systematic searches were conducted in PubMed, Scopus, and Web of Science. Clinical remission was considered as the primary endpoint. Pairwise meta-analyses were performed for the randomized controlled studies (Mantel Haenszel, random effects model). Proportion meta-analyses, accounting for weighted pooled rates reported in the interventional studies, were conducted using the mixed effects model. Subgroup analyses considering the type of stool, donor type, and disease subtype were also performed. Cumulative meta-analyses to assess further needs of evidence were conducted.

**Results:**

Sixty studies were included, from which 36 could be synthesized in the quantitative analyses. Pairwise meta-analyses of six controlled trials showed significant differences in favor of fecal microbiota transplantation compared with placebo (clinical remission: RR 1.70 [95% CI 1.12, 2.56]; clinical response: RR 1.68 [95% CI 1.04, 2.72]). An overall clinical remission of 37%, overall clinical response of 54%, and a prevalence of 29% of adverse events were found for the interventional studies. Frozen fecal material and universal donors were related to better efficacy outcomes. In addition, Crohn’s disease patients seemed to benefit more from the procedure.

**Conclusions:**

The comparative analyses demonstrated that frozen fecal material from universal donors may be related to a higher rate of clinical remission, especially for Crohn’s disease.

## Introduction

Inflammatory bowel disease (IBD) affects about 15% of the worldwide population [1]. Clinical signs and symptoms of IBD comprise abdominal pain, anemia, diarrhea, fecal urgency, nausea, chronic fatigue, weight loss, and gastrointestinal bleeding, producing vitamin deficiencies. Studies evaluating burden of diseases highlight the negative impact of active IBD on the patients’ quality of life, especially concerning daily activities and well-being [2–4].

The goal of IBD treatment is to control the inflammatory process, achieving disease remission. Current therapies include corticosteroids, anti-tumor necrosis factor alpha (TNF-α) agents (e.g. vedolizumab and ustekinumab), aminosalicylates, immunomodulators (e.g. methotrexate), and surgery [5]. Despite the amount of available therapies, many patients are unresponsive to these treatments or present secondary failure during treatment. Hence, the development of new therapies and the investigation of alternative strategies are needed [6].

Fecal microbiota transplantation (FMT) arises as an alternative therapeutic strategy for the management of several gastrointestinal disorders, including IBD, since the gut inflammatory process is frequently associated with dysbiosis. FMT consists of the transfer of fecal material from a healthy donor, via the lower gastrointestinal tract or upper gastrointestinal tract, aiming to restore the individual's normal intestinal microbiota [1, 7].

The use of FMT is well established for the treatment of recurrent *Clostridium difficile* infection (CDI), with a large body of evidence proving the efficacy of the transplant for this purpose [8]. Since FMT is currently being considered as an experimental treatment for IBD [9], it is highly relevant to critically analyze the available evidence regarding this strategy, to guide clinicians. Therefore, the aim of the present systematic review was to synthesize published data on the efficacy and safety of FMT in patients with IBD.

## Materials and methods

### Ethics statement: Not applicable

A systematic review following Preferred Reporting Items for Systematic Reviews and Meta-Analyses (PRISMA) and the Joanna Briggs Institute recommendations was conducted. The search strategy and inclusion criteria were directed by the Population, Intervention, Comparator, Outcome, Study design (PICOS). Hence, we included interventional or observational studies evaluating the efficacy / effectiveness or safety of FMT in patients of any age or gender diagnosed with IBD (namely Ulcerative colitis, Crohn’s disease, and Pouchitis [10–12]), with or without a treatment comparator and reporting response, remission or adverse events. Exclusion criteria comprised articles published in non-Roman characters, studies including IBD patients in which the indication of FMT was not IBD (e.g. CDI) and studies that did not address the outcomes of interest (i.e. clinical remission, clinical response, adverse events).

Searches were performed in PubMed, Scopus, and Web of Science without limits for timeframe or language (last update April 2020). The following descriptors, combined with the Booleans AND and OR, were used: "Fecal Microbiota Transplantation"; "Fecal Microbiota"; "Foecal Microbiota"; "Faecal Microbiota"; Transplant*; encapsul*; capsul*; Fecal; Microbiota; Bacteriotherapy. Manual searches of the reference lists from the included studies were conducted. As we intended to perform a highly comprehensive search, descriptors for the study design were not included in the search strategy. The complete search strategy is available in the Supplemental Digital Content.

Two independent reviewers screened the titles and abstracts to identify irrelevant records. Then, full-text articles were appraised. In case of disagreements, a third reviewer acted as referee. A standardized form to collect data on the general characteristics (e.g. author, country) and clinical outcomes of the included studies was used. The methodological quality of included randomized controlled trials (RCTs) was assessed using the Cochrane risk-of-bias tool for randomized trials [13], and the quasi-experimental studies were appraised using the Newcastle-Ottawa Scale (NOS) [14].

The primary endpoint outcome was clinical remission, and the secondary outcomes were clinical response and any adverse event. For studies with comparative arms, a pairwise meta-analysis was performed using Review Manager, version 5.1 (The Nordic Cochrane Centre, The Cochrane Collaboration, Copenhagen, Sweden). Results were expressed as risk ratios (RR) with a 95% confidence interval (CI). The Mantel Haenszel statistic and the random-effects model were applied. *P*-values lower than 0.05 (two-tailed) were considered indicative of a statistically significant difference between groups.

For both the comparative studies and single arm studies, a proportion meta-analysis of weighted pooled rates with a 95% CIs were calculated for each outcome, using a mixed effects model. In addition, subgroup analyses considering the type of stool (fresh or frozen), type of donor (relative/acquaintance or universal [i.e., a donor not related to the patient]), and type of IBD (Crohn or ulcerative colitis) were performed using Comprehensive Meta-Analysis (CMA) (Version 2.0, Biostat, Englewood, NJ). Both for the pairwise and prevalence meta-analyses, between-trial heterogeneity was assessed using the inconsistency relative index, I^2^ (I^2^ > 50% indicates high and significant heterogeneity) [15].

To evaluate whether further clinical trials are needed to provide more robust evidence on the effects of FMT for patients with IBD, a cumulative meta-analysis was performed for the primary outcome (clinical remission) using Comprehensive Meta-Analysis (CMA) (Version 2.0, Biostat, Englewood, NJ).

To assess the accuracy of the results and the effect of individual studies on data heterogeneity, sensitivity analyses were performed by the hypothetical and sequential removal of studies from the meta-analyses. No study was permanently removed. Funnel plot was applied to evaluate publication bias, along with Egger’s test, for which p-value less than 0.05 indicates potential publication bias [16].

## Results

**Fig 1:**
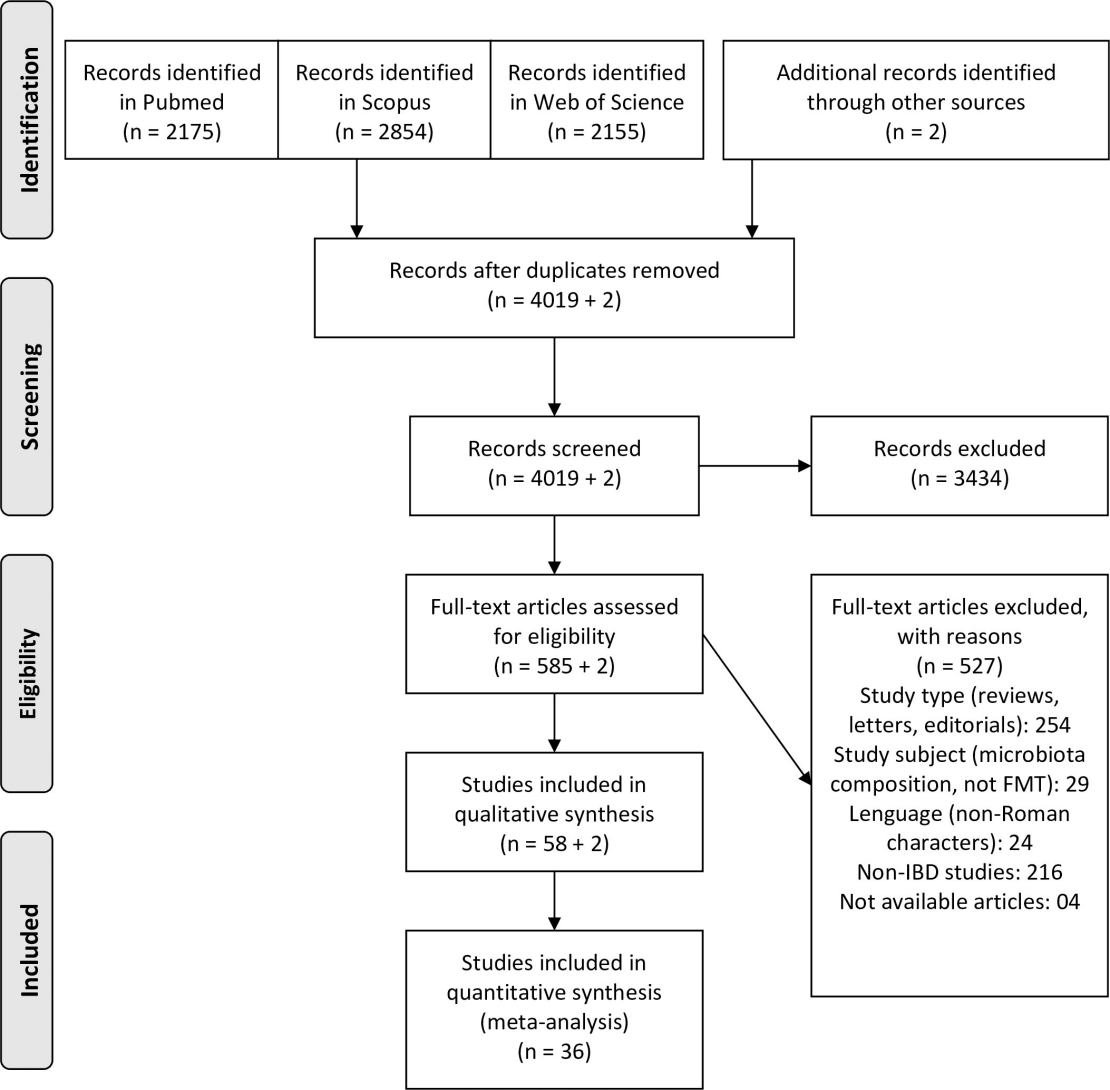
Preferred Reporting Items for Systematic Reviews and Meta-Analyses (PRISMA) 2009 flow diagram.

The searches in the databases yielded 4,019 records after duplicates were removed. During the screening of titles and abstracts, 3,434 were considered irrelevant records, and, subsequently, 585 studies were assessed after full text appraisal. Two additional records were identified from manual searches, resulting in a total of 60 studies for the qualitative synthesis, with 36 that could be analyzed in the quantitative synthesis (Fig 1). These 60 studies included 27 quasi-experimental trials (interventional studies without randomization or without a control group), 9 RCTs, 5 cohort studies, 5 case series, and 14 case reports. The list of the 60 included references is provided in the Supplemental Digital Content (S1 Table). The 36 records included in the quantitative synthesis comprised all the quasi-experimental studies [17–44] and RCTs [45–53]. Data on these studies are provided in Table 1. The included RCTs presented an overall moderate risk of bias (S1 Fig). The mean NOS score for the quasi-experimental studies was 6 (ranging from 6 to 8).

**Table 1:** Included studies of fecal microbiota transplantation for inflammatory bowel diseases.

Author	Study type	Country	Number of Patients	FMT indication	Donor	Type of stool	Adminsitration route^†^
Kump, 2018 [17]	Quasi-experimental	Austria	27	Ulcerative colitis	not specifiedǂ	fresh	upper or lower
Goyal, 2018 [18]	Quasi-experimental	USA	21	Any IBD	relative or acquaintance	fresh	upper or lower
Uygun, 2017 [19]	Quasi-experimental	Turkey	30	Ulcerative colitis	not specifiedǂ	fresh	lower
Nishida, 2017 [20]	Quasi-experimental	Japan	41	Ulcerative colitis	relative or acquaintance	fresh	lower
Karolewska-Bochenek, 2017 [21]	Quasi-experimental	Poland	10	Any IBD	universal	frozen	upper
Jacob, 2017 [22]	Quasi-experimental	USA	20	Ulcerative colitis	universal	fresh	lower
Ishikawa, 2017 [23]	Quasi-experimental	Japan	41	Ulcerative colitis	relative or acquaintance	fresh	lower
Fang, 2017 [24]	Quasi-experimental	China	5	Any IBD	relative or acquaintance	fresh	upper or lower
Zhang, 2016 [25]	Quasi-experimental	China	28	Ulcerative colitis	NR	fresh	upper
Vermeire, 2016 [26]	Quasi-experimental	Belgium	14	Any IBD	relative or acquaintance	fresh	upper or lower
Vaughn, 2016 [27]	Quasi-experimental	USA	19	Crohn's disease	universal	frozen	lower
Wei, 2015 [28]	Quasi-experimental	China	14	Any IBD	universal	fresh	upper or lower
Damman, 2015 [29]	Quasi-experimental	USA	7	Ulcerative colitis	relative or acquaintance	fresh	lower
Kunde, 2013 [30]	Quasi-experimental	USA	10	Ulcerative colitis	relative or acquaintance	fresh	lower
Kump, 2013 [31]	Quasi-experimental	Austria	6	Ulcerative colitis	universal	fresh	lower
Landy, 2015 [32]	Quasi-experimental	United Kingdom	8	Chronic pouchitis	not specifiedǂ	fresh	upper
Suskind, 2015a [33]	Quasi-experimental	USA	9	Crohn's disease	relative or acquaintance	NR	upper
Suskind, 2015b [34]	Quasi-experimental	USA	4	Ulcerative colitis	NR	NR	upper
Sood, 2019 [35]	Quasi-experimental	India	41	Ulcerative colitis	universal	fresh	lower
Wang, 2018 [36]	Quasi-experimental	China	139	Crohn's disease	not specifiedǂ	fresh or frozen	upper
Mizuno, 2017 [44]	Quasi-experimental	Japan	10	Ulcerative colitis	relative or acquaintance	fresh	lower
Adler, 2019 [37]	Quasi-experimental	USA	13	Ulcerative colitis	universal	capsules	oral
Xiang, 2019 [38]	Quasi-experimental	China	174	Crohn's disease	not specifiedǂ	fesh	upper or lower
Gutin, 2019 [39]	Quasi-experimental	USA	10	Crohn's disease	universal	frozen	lower
Tian, 2019 [40]	Quasi-experimental	China	20	Ulcerative colitis	universal	fresh	upper
Selvig, 2019 [41]	Quasi-experimental	USA	18	Chronic pouchitis	universal	frozen	lower
Zou, 2019 [42]	Quasi-experimental	China	15	Any IBD	relative or acquaintance	fresh	upper
Cold, 2019 [43]	Quasi-experimental	Denmark	7	Ulcerative colitis	universal	capsules	oral
Paramsothy, 2017 [45]	RCT	Australia	81	Ulcerative colitis	universal	frozen	lower
Wei, 2016 [46]	RCT	China	20	Ulcerative colitis	universal	fresh	lower
Rossen, 2015 [47]	RCT	Netherlands	48	Ulcerative colitis	not specifiedǂ	fresh	upper
Moayyedi, 2015 [48]	RCT	Canada	75	Ulcerative colitis	universal	fresh or frozen	lower
Costello, 2019 [49]	RCT	Australia	73	Ulcerative colitis	universal	frozen	lower
Herfarth, 2019 [50]	RCT	USA	6	Chronic pouchitis	universal	frozen and capsules	upper and oral
Yang, 2019 [51]	RCT	China	27	Crohn's disease	not specifiedǂ	fesh	upper or lower
Sokol, 2020 [52]	RCT	France	17	Crohn's disease	universal	fresh	lower
Sood, 2019b [53]	RCT	India	61	Ulcerative colitis	universal	fresh or frozen	lower

RCT, randomized controlled trial. NR, not reported.

† upper = nasogastric tube; lower = colonoscopy or enema.

ǂ not specified includes relative, acquaintance and universal donors.

### Pairwise meta-analyses

Six of the nine included RCTs presented comparable data and were assessed in two meta-analyses, one for clinical remission and one for clinical response [45, 47–49, 52, 53]. For both outcomes, statistically significant differences were observed favoring FMT over placebo (355 patients—clinical remission: RR 1.70 [95% CI 1.12, 2.56], I^2^ = 45%; clinical response: RR 1.68 [95% CI 1.04, 2.72], I^2^ = 55%). The forest plots are presented in the Supplemental Digital Content (S2 and S3 Figs). In the sensitivity analyses, the study from Rossen et al. [47] was responsible for increasing heterogeneity in the clinical response meta-analysis. After removing this study, the I^2^ value dropped to 0%, and more pronounced results favoring FMT over placebo were observed (clinical response: RR 2.13 [95% CI 1.45, 3.12]). The nasogastric route was used (upper gastrointestinal tract) in the study by Rossen et al., [47] whereas the other studies used colonoscopy and enema (lower gastrointestinal tract) [45, 48, 49]. In addition, donors of the fecal material in the Rossen et al. study consisted of relatives, acquaintances, and universal donors [47], while all fecal samples in the other three studies were collected from universal donors [45, 48, 49]. No other aspects that could explain the heterogeneity between the studies were observed. For the clinical remission analysis, no study was identified as responsible for the heterogeneity.

### Proportions meta-analyses

Three outcomes were evaluated in the prevalence meta-analyses: clinical remission, clinical response, and any adverse event.

#### Clinical remission

The analysis of the 24 quasi-experimental studies assessing clinical remission [17–39, 42, 43] led to a clinical remission prevalence of 32.3% [95% CI 23.1–43.1], while the nine RCTs [45–53] resulted in a rate of 46.0% [95% CI 31.4–61.4]. The overall remission rate was 37.0% [95% CI 28.8–45.9], with no significant difference between these two study designs (*p* = 0.144).

The stool type subgroup analysis revealed clinical remission rates of 29.1% for fresh fecal material [17–20, 22–26, 28–32, 35, 38, 42, 46, 47, 51, 52], 44.2% for frozen fecal material [21, 27, 39, 45, 49, 50], 57.2% when both types of stool were used [36, 48, 53], and 66.5% for capsules [37, 43], with a statistical difference observed among the groups (*p* = 0.036). Two articles did not report the type of stool used for the FMT [33, 34] (Table 2, S4 Fig). A statistical difference was observed when only comparing studies that reported the use of fresh, frozen fecal material and capsules separately (*p* = 0.010).

**Table 2:** Efficacy of fecal microbiota transplantation by subgroups.

Subgroup/Outcome	Number of Studies (N)	Clinical remission		Number of Studies (N)	Clinical response	
		Event rate (95% CI)	*p*-value		Event rate (95% CI)	*p*-value
**Type of stool**			0.036*			0.042*
Fresh	21	29.1% (20.3–39.9)		21	52.1% (41.4–62.7)	
Frozen	6	44.2% (33.9–55.0)		7	52.8% (41.6–63.8)	
Fresh or frozen	3	57.2% (25.9–83.6)		2	56.6% (26.1–82.8)	
Capsules	2	66.5% (42.7%-84.2%)		2	94.9% (71.3%-99.3%)	
Not reported	2	-		1	-	
**Donor type**			0.029*			0.197
Relative/acquaintances	9	24.8% (11.5–45.4)		8	37.1% (21.5–56.0)	
Universal	16	45.1% (33.3–57.6)		16	59.7% (47.5–70.8)	
Not specifiedǂ	7	36.4% (21.0–55.2)		7	58.9% (43.9–72.4)	
Not reported	2	-		2	-	
**IBD subtype**			0.152			0.515
Ulcerative colitis	22	35.0% (26.0–45.2)		22	54.6% (44.0–64.8)	
Crohn's disease	10	47.6% (30.9–64.9)		7	57.9% (41.8–72.5)	
Chronic pouchitis	2	7.4% (1.0–38.2)		3	21.8% (3.7–66.8)	
Any IBD	3	41.0% (2.7–94.7)		3	58.1% (15.0–91.5)	

IBD, inflammatory bowel disease.

*Statistically significant.

ǂ Not specified includes relative, acquaintance and universal donors.

In the sub-analysis by donor type, seven studies did not specify the source of stool [17, 19, 32, 36, 38, 47, 51], showing a remission of 36.4%. In nine studies [18, 20, 23, 24, 26, 29, 30, 33, 42], the donors of fecal material were relatives or acquaintances, resulting in a clinical remission of 24.8%. Universal donors provided fecal material in 16 studies [21, 22, 27, 28, 31, 35, 37, 39, 43, 45, 46, 48–50, 52, 53], which led to a remission of 45.1%. Two articles [25, 34] did not report the source of fecal material (Table 2, S5 Fig). Although a statistically significant difference was observed between the donor type groups (*p* = 0.029), no statistical difference was found (*p* = 0.089) when considering only studies that used fecal material from relatives or acquaintances compared with universal donors.

Patients with Crohn’s disease presented a remission rate of 47.6% [18, 21, 27, 33, 36, 38, 39, 42, 51, 52], whereas patients with ulcerative colitis showed a remission rate of 35.0% [17–23, 25, 29–31, 34, 35, 37, 42, 43, 45–49, 53]. Three studies assessed FMT without discriminating the type of IBD [24, 26, 28], resulting in a remission of 41.0%, and two studies [32, 50] reported a 7.4% remission rate in patients with chronic pouchitis. No statistically significant difference was observed for the IBD type subgroup analysis (*p* = 0.152) (Table 2, S6 Fig). When considering only studies that evaluated patients with Crohn’s disease and ulcerative colitis separately, no statistically significant difference was observed (*p* < 0.214).

Sensitivity analysis showed that the results remained unchanged with the hypothetical removal of one study (S7 Fig).

#### Clinical response

A total of 26 quasi-experimental studies [17–33, 35–41, 43, 44] and seven RCTs [45–51] were pooled, resulting in a response of 53.5% [95% CI 43.1–63.5] and 54.4% [42.6–65.7], respectively. The overall pooled response was 53.8% [95% CI 46.0–61.5]. Once both types of studies provided similar results (*p* = 0.910), they were aggregated for the subgroup analyses.

Patients who received fresh fecal material (reported in 21 studies [17–20, 22–26, 28–32, 35, 38, 40, 44, 46, 47, 51]) exhibited a clinical response of 52.1%, while individuals who received frozen fecal material (reported in seven studies [21, 27, 39, 41, 45, 49, 50]) presented a response of 52.8%. In two studies [36, 48], both fresh and frozen fecal material were administered in IBD patients, resulting in an overall clinical response of 56.6%. Two studies [37, 43] reported the use of capsules, with a clinical response of 94.9%. One study did not report the stool type [33]. Statistically significant difference was observed between the type of stool groups (*p* = 0.042) (Table 2, S8 Fig).

When the fecal material was provided by relatives or acquaintances [18, 20, 23, 24, 26, 29, 30, 44], the pooled clinical response was 37.1%, whereas patients who received fecal material from universal donors [21, 22, 27, 28, 31, 35, 37, 39–41, 43, 45, 46, 48–50] exhibited a response of 59.7%. Seven studies did not specify the type of donor, resulting in a prevalence of 58.9% for the clinical response [17, 19, 32, 36, 38, 47, 51]. Two studies did not report the donor type [25, 34]. No statistically significant difference was observed between the type of donor groups for this analysis (*p* = 0.197) (Table 2, S9 Fig).

Three studies did not distinguish the IBD subtype, resulting in a clinical response of 58.1% [24, 26, 28]. Seven studies [18, 21, 27, 36, 38, 39, 51] included patients with Crohn’s disease, resulting in a 57.9% clinical response, whereas ulcerative colitis patients (evaluated in 22 studies [17–23, 25, 29–31, 34, 35, 37, 40, 43–49]) exhibited a response of 54.6%. Three studies [32, 41, 50] assessed patients with chronic pouchitis, resulting in a response of 21.8%. No statistical difference was observed between the IBD groups (*p* = 0.515) (Table 2, S10 Fig).

The sensitivity analysis showed that the results did not change with the hypothetical removal of one study (S11 Fig).

#### Any adverse event

Sixteen quasi-experimental studies [18, 19, 21, 23–25, 28, 31–34, 36–38, 40, 42, 43] (the two articles of Suskind et al., were grouped for this outcome) and four RCTs [45–47, 50] were included in the analysis, resulting in a frequency of 26.9% [95% CI 16.5–40.6] and 48.2% [95% CI 15.4–82.6], respectively, for any adverse event. The overall frequency of adverse events was 29.2% [95% CI 18.8–42.5]. Both study designs provided similar results (*p* = 0.295), therefore they were gathered for the subgroup analyses.

In studies where fresh fecal material was transplanted [18, 19, 23–25, 28, 31, 32, 38, 40, 42, 46, 47], the frequency of any adverse event was 30.2%, whereas 43.2% was observed in articles using frozen fecal material [21, 45, 50] and 22.6% was observed in studies using capsules [37, 43]. In one study, both fresh and frozen material were used, resulting in a prevalence of 14.4% for any adverse event [36]. The studies by Suskind et al. did not report the stool type [33, 34]. A statistically significant difference between stool type groups was observed (*p* = 0.033) (Table 3, S12 Fig).

**Table 3:** Safety of fecal microbiota transplantation by subgroups.

Subgroup/Outcome	Studies (N)	Adverse events	
		Event rate (95% CI)	*p*-value
**Type of stool**			0.033*
Fresh	13	30.2% (15.9–49.7)	
Frozen	3	43.2% (9.6–84.5)	
Fresh or frozen	1	14.4% (9.5–21.3)	
Capsules	2	22.6% (4.0%-67.3%)	
Not reported	1	-	
**Donor type**			0.222
Relative/acquaintances	4	48.0% (21.1–76.0)	
Universal	9	26.5% (12.1–48.6)	
Not specifiedǂ	5	22.9% (6.7–55.0)	
Not reported	2	-	
**IBD subtype**			0.047*
Ulcerative colitis	11	36.9% (21.5–55.6)	
Crohn's disease	3	5.8% (1.2–23.5)	
Chronic pouchitis	2	29.9% (10.1–61.7)	
Any IBD	5	44.3% (25.9–64.3)	

IBD, inflammatory bowel disease.

*Statistically significant.

ǂ Not specified includes relative, acquaintance and universal donors.

For patients who received fecal material from relatives or acquaintances (four studies [18, 23, 24, 42]) the prevalence of any adverse event was 48.0%, while patients receiving fecal material from universal donors (assessed in nine studies [21, 28, 31, 37, 40, 43, 45, 46, 50]) presented a prevalence of 26.5% for any adverse event. Five studies [19, 32, 36, 38, 47] did not specify the donor type, resulting in a prevalence of 22.9% for any adverse event. Two studies did not report the donor type [25, 34]. No statistically significant difference was observed between donor type groups (*p* = 0.222) (Table 3, S13 Fig).

Patients with Crohn’s disease exhibited a prevalence of 5.8% [36, 38, 42], whereas 36.9% was observed in individuals with ulcerative colitis [19, 23, 25, 31, 37, 40, 42, 43, 45–47] for any adverse event. For studies that did not specify the IBD subtype, the occurrence of any adverse event was 44.3% [18, 21, 24, 28, 33, 34]. Two studies investigated FMT for chronic pouchitis, with a prevalence of 29.9% for any adverse event [32, 50]. A statistically significant difference was observed between IBD type subgroups (*p* = 0.047) (Table 3, S14 Fig).

The sensitivity analysis showed that the results remained unchanged with the hypothetical removal of one study (S15 Fig).

The PRISMA checklist for the present systematic review is presented in S2 Table.

### Cumulative meta-analysis

The studies included in the cumulative meta-analysis were published between 2013 and 2020. The articles that reported the use of capsules [37, 43] were excluded from this analysis since they exhibited pronouncedly higher frequencies of clinical remission. Cumulative meta-analysis shows that the proportion of clinical remission increased over the years and has not stabilized yet (S16 Fig). The funnel plot does not indicate potential publication bias (S17 Fig), which is confirmed by the Egger’s test (p-value = 0.25).

## Discussion

FMT for the management of patients with IBD demonstrated a response rate of 53.8% with a complete remission of 37%. The administration of frozen fecal material produced better results in terms of clinical remission when compared to fresh material. A randomized controlled trial conducted in Canada compared frozen and fresh FMT in patients with recurrent CDI concluding that the efficacy was similar for both types of stool (75.0% in the frozen FMT group and 70.3% in the fresh FMT group—modified intention to treat analysis) [54]. Our results produced positive evidence towards the use of frozen fecal material, which is coincident with some previous studies [55, 56]. Nevertheless, this result should be interpreted with caution, since in all included studies the frozen fecal material was provided by universal donors, which may produce greater microbial diversity in the recipient enhancing the efficacy of the procedure [57]. In addition, for the studies that mentioned the stool processing, the freezing methods were slightly different, with protocols varying in the temperature of storage of the fecal material (-80° C [27, 45, 49] or -20° C [21, 39]). Frozen FMT offers several advantages, including immediate availability of the fecal material, cost-savings associated with the less donor screenings required, and the lower structural requirements of the practice setting where the transplantation is performed [54]. In addition, it is important to notice that the freezing and thawing process do not significantly alter the viable microbiota composition [58].

Although no significant difference was found when comparing relatives/acquaintances with universal donors, our results pointed to higher rates of clinical remission and clinical response when the fecal material was provided by universal donors. A potential reason of the lack of significance could be the large confidence intervals of the pooled proportions in relative/acquaintance and universal donors. Previous studies also showed no significant differences in outcomes between FMT from universal and from patient-identified donors [59]. These results reinforce the universal donor model, which promotes a cost-effective access to FMT [60].

Despite the significant difference observed between the type of stool groups in the adverse events assessment, pointing to a higher occurrence with frozen fecal material, this result should be interpreted with caution, since most of included studies do not standardize the adverse events report, which may generate bias. A similar situation was observed for the type of IBD, with a very small proportion of adverse events in patients with Crohn’s disease. Hence, it is not possible to definitely infer that the stool characteristics and the type of IBD influence the occurrence of adverse events related to the procedure. No difference was obtained when assessing adverse events grouped by the type of donor. Most adverse events reported in the studies were mild and included diarrhea, abdominal pain, nausea, flatulence, and fever, which ceased within 24 hours after the transplant.

FMT has been widely investigated for other gastrointestinal disorders besides CDI, but few systematic reviews have been published indicating that this procedure is effective and safe for IBD management [61–66]. In 2012, Anderson et al. conducted a systematic review evaluating FMT in IBD patients wherein no meta-analytical calculations and no controlled trial were included. Despite the weak evidence available at the time of that study, their results pointed to the potential effectiveness and safety of FMT for the management of IBD [66]. Our pairwise meta-analyses support significant differences favoring FMT for clinical remission and clinical response. Nevertheless, there are still a reduced number of comparative studies assessing FMT for IBD. This may be the reason for the over-precautious position of some consensus that limited the use of FMT for IBD, and other non-CDI gastrointestinal disorders, to research settings only [8, 67].

Narula et al. published a systematic review with meta-analysis, in 2017, evaluating FMT only for active ulcerative colitis. The authors gathered high-quality RCTs to assess clinical remission and endoscopic remission or response, including only four studies in their meta-analysis. Their findings demonstrated a higher clinical remission combined with endoscopic remission in the FMT group when compared to placebo, with no significant difference between FMT and placebo for serious adverse events [64].

A recent study compared the efficacy and safety of biological agents, tofacitinib, and FMT in ulcerative colitis through a systematic review and network meta-analysis of RCTs. The results of the study showed that all evaluated treatments were more effective than the placebos, with no statistical difference in the efficacy of biological agents, tofacitinib, and FMT. In addition, with the exception of infliximab, no active treatment increased the occurrence of adverse events when compared to the placebos. In conclusion, the authors point FMT and tofacitinib as promising alternatives for ulcerative colitis management [68].

Dang et al. performed a systematic review with meta-analysis comparing FMT with mixed probiotics therapy in ulcerative colitis patients. The authors included RCTs in their analysis showing that all treatments were superior to placebo with no increased risk of adverse events. In addition, no statistical difference was observed between FMT and the probiotics in terms of clinical efficacy. Despite these promising results, the authors point to many unresolved issues regarding the clinical application of these alternatives, suggesting that more randomized controlled trials are needed [69].

Our results were also promising towards FMT and we additionally performed several subgroup analyses regarding the type of stool, type of donor and IBD subtype. The concerns raised by the aforementioned studies on the need of more clinical trials to confirm FMT effects are in line with the results of our cumulative meta-analysis, which shows that the proportion of clinical remission has not yet reached stability. This means that further studies would potentially increment the evidence on FMT for the treatment of IBD. It is observed that the proportion of clinical remission increased over the evaluated years, probably due to improvements in the protocols of FMT, especially concerning more stringent criteria for selection and screening of donors [70]. Despite de need for more studies assessing FMT efficacy, no publication bias was detected in our analysis, revealing the comprehensiveness of our search and the robustness of our results.

In 2017, Paramsothy et al. conducted a broad systematic review with meta-analysis to assess the effectiveness and safety of FMT in IBD patients [62]. Our results are in consonance with their findings for clinical remission and clinical response in Crohn’s disease (52% clinical remission in the Paramsothy et al. study versus 47.6% in our study) and ulcerative colitis (33% versus 35% for clinical remission, respectively; 52% vs 54.6% for clinical response, respectively). Nevertheless, unlike Paramsothy et al., we preferred not to include conference abstracts to gather more reliable evidence. These authors conducted some subgroup analyses, however, their results for donor type were uncertain due to the small number of studies. In contrast to Paramsothy et al., the studies included in our systematic review allowed quantitative analyses of adverse events, demonstrating the benefits of FMT for patients with Crohn’s disease and ulcerative colitis [62].

The Third European Evidence-based Consensus on Diagnosis and Management of Ulcerative Colitis, published by the European Crohn’s and Colitis Organisation (ECCO), recognizes the encouraging results from RCTs evaluating the ability of FMT to achieve clinical remission in patients with active ulcerative colitis. Nevertheless, the ECCO guideline highlights the need for additional studies to identify the best strategy, taking into consideration the administration route and donor type [71]. Our study pointed to some crucial elements in FMT protocols to optimize the efficacy and safety in IBD patients. Conversely, the 3rd European Evidence-based Consensus on the Diagnosis and Management of Crohn’s Disease does not mention FMT as a potential alternative for the treatment of this condition [72]. Notwithstanding, our results demonstrated that patients with Crohn’s disease seem to achieve even better outcomes with the FMT than ulcerative colitis patients.

The present systematic review has some limitations. First, only interventional studies were analyzed, so further analysis on observational studies should be conducted to add information on the effectiveness of FMT in patients with IBD. Second, the analysis on adverse events should be interpreted with caution due to potential bias, since several included studies did not report specific events nor provided a detailed information on them. Finally, attention should be taken in the analysis on the type of stool, since this variable may be a confounding. The analysis and the literature show that stool from universal donors may provide better results with FMT procedure, and the included studies with frozen stool used universal donors.

In conclusion, our systematic review demonstrated the positive effects of FMT for IBD management with significant differences compared to placebo. Additionally, several high-quality, non-randomized, quasi-experimental studies showed that FMT is a safe alternative with promising remission and response rates. We also found that the use of frozen fecal material from universal donors may be associated with better efficacy outcomes in IBD patients, especially those with Crohn’s disease.

## Supporting information

S1 Checklist PRISMA 2009 checklist. (DOC)

S1 Table List of the 45 included references. (DOCX)

S2 Table PRISMA checklist. (DOCX)

S1 Fig Risk of bias graph of included RCTs. (DOCX)

S2 Fig Forest plot for clinical remission. (DOCX)

S3 Fig Forest plot for clinical response. (DOCX)

S4 Fig Clinical remission—subgroup analysis by stool type. (DOCX)

S5 Fig Clinical remission—subgroup analysis by donor type. (DOCX)

S6 Fig Clinical remission—subgroup analysis by IBD subtype. (DOCX)

S7 Fig Sensitivity analysis for clinical remission. (DOCX)

S8 Fig Clinical response—subgroup analysis by stool type. (DOCX)

S9 Fig Clinical response—subgroup analysis by donor type. (DOCX)

S10 Fig Clinical response—subgroup analysis by IBD subtype. (DOCX)

S11 Fig Sensitivity analysis for clinical response. (DOCX)

S12 Fig Any adverse event—subgroup analysis by stool type. (DOCX)

S13 Fig Any adverse event—subgroup analysis by donor type. (DOCX)

S14 Fig Any adverse event—subgroup analysis by IBD subtype. (DOCX)

S15 Fig Sensitivity analysis for any adverse event. (DOCX)

S16 Fig Cumulative meta-analysis for clinical remission. (DOCX)

S17 Fig Funnel plot for assessment of potential publication bias. (DOCX)

## References

[1] Sunkara T, Rawla P, Ofosu A, Gaduputi V (2018) Fecal microbiota transplant—a new frontier in inflammatory bowel disease. J Inflamm Res 11: 321–328. 10.2147/JIR.S176190 30214266 PMC6124474

[2] Fabisiak N, Fabisiak A, Watala C, Fichna J (2017) Fat-soluble Vitamin Deficiencies and Inflammatory Bowel Disease: Systematic Review and Meta-Analysis. J Clin Gastroenterol 51: 878–889. 10.1097/MCG.0000000000000911 28858940

[3] Yarlas A, Rubin DT, Panes J, Lindsay JO, Vermeire S, et al (2018) Burden of Ulcerative Colitis on Functioning and Well-being: A Systematic Literature Review of the SF-36(R) Health Survey. J Crohns Colitis 12: 600–609. 10.1093/ecco-jcc/jjy024 29718244

[4] Pihl-Lesnovska K, Hjortswang H, Ek AC, Frisman GH (2010) Patients' perspective of factors influencing quality of life while living with Crohn disease. Gastroenterol Nurs 33: 37–44; quiz 45–36. 10.1097/SGA.0b013e3181cd49d0 20145449

[5] Vindigni SM, Zisman TL, Suskind DL, Damman CJ (2016) The intestinal microbiome, barrier function, and immune system in inflammatory bowel disease: a tripartite pathophysiological circuit with implications for new therapeutic directions. Therap Adv Gastroenterol 9: 606–625. 10.1177/1756283X16644242 27366227 PMC4913337

[6] Weisshof R, El Jurdi K, Zmeter N, Rubin DT (2018) Emerging Therapies for Inflammatory Bowel Disease. Adv Ther 35: 1746–1762. 10.1007/s12325-018-0795-9 30374806 PMC6224002

[7] Heath RD, Cockerell C, Mankoo R, Ibdah JA, Tahan V (2018) Fecal microbiota transplantation and its potential therapeutic uses in gastrointestinal disorders. North Clin Istanb 5: 79–88. 10.14744/nci.2017.10692 29607440 PMC5864716

[8] Cammarota G, Ianiro G, Tilg H, Rajilic-Stojanovic M, Kump P, et al (2017) European consensus conference on faecal microbiota transplantation in clinical practice. Gut 66: 569–580. 10.1136/gutjnl-2016-313017 28087657 PMC5529972

[9] Moore T, Rodriguez A, Bakken JS (2014) Fecal microbiota transplantation: a practical update for the infectious disease specialist. Clin Infect Dis 58: 541–545. 10.1093/cid/cit950 24368622

[10] van der Sloot KWJ, Amini M, Peters V, Dijkstra G, Alizadeh BZ (2017) Inflammatory Bowel Diseases: Review of Known Environmental Protective and Risk Factors Involved. Inflamm Bowel Dis 23: 1499–1509. 10.1097/MIB.0000000000001217 28777099

[11] Maaser C, Sturm A, Vavricka SR, Kucharzik T, Fiorino G, et al (2018) ECCO-ESGAR Guideline for Diagnostic Assessment in IBD Part 1: Initial diagnosis, monitoring of known IBD, detection of complications. J Crohns Colitis. 10.1093/ecco-jcc/jjy113 30137275

[12] Mahadevan U, Sandborn WJ (2003) Diagnosis and management of pouchitis. Gastroenterology 124: 1636–1650. 10.1016/s0016-5085(03)00325-1 12761722

[13] Higgins JP, Altman DG, Gotzsche PC, Juni P, Moher D, et al (2011) The Cochrane Collaboration's tool for assessing risk of bias in randomised trials. BMJ 343: d5928 10.1136/bmj.d5928 22008217 PMC3196245

[14] Wells GA, Shea B, O'Connell D, Peterson J, Welch V, Losos M, et al The Newcastle-Ottawa Scale [NOS] for assessing the quality of nonrandomised studies in meta-analyses. http://www.ohri.ca/programs/clinical_epidemiology/oxford.asp. Accessed February 2, 2019.

[15] Huedo-Medina TB, Sanchez-Meca J, Marin-Martinez F, Botella J (2006) Assessing heterogeneity in meta-analysis: Q statistic or I2 index? Psychol Methods 11: 193–206. 10.1037/1082-989X.11.2.193 16784338

[16] van Enst WA, Ochodo E, Scholten RJ, Hooft L, Leeflang MM (2014) Investigation of publication bias in meta-analyses of diagnostic test accuracy: a meta-epidemiological study. BMC Med Res Methodol 14: 70 10.1186/1471-2288-14-70 24884381 PMC4035673

[17] Kump P, Wurm P, Grochenig HP, Wenzl H, Petritsch W, et al (2018) The taxonomic composition of the donor intestinal microbiota is a major factor influencing the efficacy of faecal microbiota transplantation in therapy refractory ulcerative colitis. Aliment Pharmacol Ther 47: 67–77. 10.1111/apt.14387 29052237 PMC5765501

[18] Goyal A, Yeh A, Bush BR, Firek BA, Siebold LM, et al (2018) Safety, Clinical Response, and Microbiome Findings Following Fecal Microbiota Transplant in Children With Inflammatory Bowel Disease. Inflamm Bowel Dis 24: 410–421. 10.1093/ibd/izx035 29361092

[19] Uygun A, Ozturk K, Demirci H, Oger C, Avci IY, et al (2017) Fecal microbiota transplantation is a rescue treatment modality for refractory ulcerative colitis. Medicine (Baltimore) 96: e6479. 28422836 10.1097/MD.0000000000006479 PMC5406052

[20] Nishida A, Imaeda H, Ohno M, Inatomi O, Bamba S, et al (2017) Efficacy and safety of single fecal microbiota transplantation for Japanese patients with mild to moderately active ulcerative colitis. J Gastroenterol 52: 476–482. 10.1007/s00535-016-1271-4 27730312

[21] Karolewska-Bochenek K, Grzesiowski P, Banaszkiewicz A, Gawronska A, Kotowska M, et al (2018) A Two-Week Fecal Microbiota Transplantation Course in Pediatric Patients with Inflammatory Bowel Disease. Adv Exp Med Biol 1047: 81–87. 10.1007/5584_2017_123 29151253

[22] Jacob V, Crawford C, Cohen-Mekelburg S, Viladomiu M, Putzel GG, et al (2017) Single Delivery of High-Diversity Fecal Microbiota Preparation by Colonoscopy Is Safe and Effective in Increasing Microbial Diversity in Active Ulcerative Colitis. Inflamm Bowel Dis 23: 903–911. 10.1097/MIB.0000000000001132 28445246 PMC6159890

[23] Ishikawa D, Sasaki T, Osada T, Kuwahara-Arai K, Haga K, et al (2017) Changes in Intestinal Microbiota Following Combination Therapy with Fecal Microbial Transplantation and Antibiotics for Ulcerative Colitis. Inflamm Bowel Dis 23: 116–125. 10.1097/MIB.0000000000000975 27893543

[24] Fang Y, Chen J, Yu J, Luo Y, Lou J (2017) The Preliminary Investigation of Faecal Microbiota Transplantation for Paediatric Recurrent Chronic Bowel Diseases and Literature Review. Hong Kong Journal of Paediatrics 22: 199–203.

[25] Zhang T, Cui B, Li P, He Z, Long C, et al (2016) Short-Term Surveillance of Cytokines and C-Reactive Protein Cannot Predict Efficacy of Fecal Microbiota Transplantation for Ulcerative Colitis. PLoS One 11: e0158227 10.1371/journal.pone.0158227 27347881 PMC4922664

[26] Vermeire S, Joossens M, Verbeke K, Wang J, Machiels K, et al (2016) Donor Species Richness Determines Faecal Microbiota Transplantation Success in Inflammatory Bowel Disease. J Crohns Colitis 10: 387–394. 10.1093/ecco-jcc/jjv203 26519463 PMC4946755

[27] Vaughn BP, Vatanen T, Allegretti JR, Bai A, Xavier RJ, et al (2016) Increased Intestinal Microbial Diversity Following Fecal Microbiota Transplant for Active Crohn's Disease. Inflamm Bowel Dis 22: 2182–2190. 10.1097/MIB.0000000000000893 27542133 PMC4995064

[28] Wei Y, Zhu W, Gong J, Guo D, Gu L, et al (2015) Fecal Microbiota Transplantation Improves the Quality of Life in Patients with Inflammatory Bowel Disease. Gastroenterol Res Pract 2015: 517597 10.1155/2015/517597 26146498 PMC4471308

[29] Damman CJ, Brittnacher MJ, Westerhoff M, Hayden HS, Radey M, et al (2015) Low Level Engraftment and Improvement following a Single Colonoscopic Administration of Fecal Microbiota to Patients with Ulcerative Colitis. PLoS One 10: e0133925 10.1371/journal.pone.0133925 26288277 PMC4544847

[30] Kunde S, Pham A, Bonczyk S, Crumb T, Duba M, et al (2013) Safety, tolerability, and clinical response after fecal transplantation in children and young adults with ulcerative colitis. J Pediatr Gastroenterol Nutr 56: 597–601. 10.1097/MPG.0b013e318292fa0d 23542823

[31] Kump PK, Grochenig HP, Lackner S, Trajanoski S, Reicht G, et al (2013) Alteration of intestinal dysbiosis by fecal microbiota transplantation does not induce remission in patients with chronic active ulcerative colitis. Inflamm Bowel Dis 19: 2155–2165. 10.1097/MIB.0b013e31829ea325 23899544

[32] Landy J, Walker AW, Li JV, Al-Hassi HO, Ronde E, et al (2015) Variable alterations of the microbiota, without metabolic or immunological change, following faecal microbiota transplantation in patients with chronic pouchitis. Sci Rep 5: 12955 10.1038/srep12955 26264409 PMC4532993

[33] Suskind DL, Brittnacher MJ, Wahbeh G, Shaffer ML, Hayden HS, et al (2015) Fecal microbial transplant effect on clinical outcomes and fecal microbiome in active Crohn's disease. Inflamm Bowel Dis 21: 556–563. 10.1097/MIB.0000000000000307 25647155 PMC4329080

[34] Suskind DL, Singh N, Nielson H, Wahbeh G (2015) Fecal microbial transplant via nasogastric tube for active pediatric ulcerative colitis. J Pediatr Gastroenterol Nutr 60: 27–29. 10.1097/MPG.0000000000000544 25162366

[35] Sood A, Mahajan R, Juyal G, Midha V, Grewal CS, et al (2019) Efficacy of fecal microbiota therapy in steroid dependent ulcerative colitis: a real world intention-to-treat analysis. Intest Res 17: 78–86. 10.5217/ir.2018.00089 30449078 PMC6361022

[36] Wang H, Cui B, Li Q, Ding X, Li P, et al (2018) The Safety of Fecal Microbiota Transplantation for Crohn's Disease: Findings from A Long-Term Study. Adv Ther 35: 1935–1944. 10.1007/s12325-018-0800-3 30328062 PMC6223988

[37] Adler E, Tabaa A, Kassam Z, Zydek M, Terdiman J, et al (2019) Capsule-Delivered Fecal Microbiota Transplant Is Safe and Well Tolerated in Patients with Ulcerative Colitis. Dig Dis Sci 64: 2452–2454. 10.1007/s10620-019-05596-5 30919208

[38] Xiang L, Ding X, Li Q, Wu X, Dai M, et al (2020) Efficacy of faecal microbiota transplantation in Crohn's disease: a new target treatment? Microb Biotechnol 13: 760–769. 10.1111/1751-7915.13536 31958884 PMC7111085

[39] Gutin L, Piceno Y, Fadrosh D, Lynch K, Zydek M, et al (2019) Fecal microbiota transplant for Crohn disease: A study evaluating safety, efficacy, and microbiome profile. United European Gastroenterol J 7: 807–814. 10.1177/2050640619845986 31316785 PMC6620877

[40] Tian Y, Zhou Y, Huang S, Li J, Zhao K, et al (2019) Fecal microbiota transplantation for ulcerative colitis: a prospective clinical study. BMC Gastroenterol 19: 116 10.1186/s12876-019-1010-4 31272391 PMC6610864

[41] Selvig D, Piceno Y, Terdiman J, Zydek M, Umetsu SE, et al (2020) Fecal Microbiota Transplantation in Pouchitis: Clinical, Endoscopic, Histologic, and Microbiota Results from a Pilot Study. Dig Dis Sci 65: 1099–1106. 10.1007/s10620-019-05715-2 31302808

[42] Zou M, Jie Z, Cui B, Wang H, Feng Q, et al (2020) Fecal microbiota transplantation results in bacterial strain displacement in patients with inflammatory bowel diseases. FEBS Open Bio 10: 41–55. 10.1002/2211-5463.12744 31622538 PMC6943227

[43] Cold F, Browne PD, Gunther S, Halkjaer SI, Petersen AM, et al (2019) Multidonor FMT capsules improve symptoms and decrease fecal calprotectin in ulcerative colitis patients while treated—an open-label pilot study. Scand J Gastroenterol 54: 289–296. 10.1080/00365521.2019.1585939 30946615

[44] Mizuno S, Nanki K, Matsuoka K, Saigusa K, Ono K, et al (2017) Single fecal microbiota transplantation failed to change intestinal microbiota and had limited effectiveness against ulcerative colitis in Japanese patients. Intest Res 15: 68–74. 10.5217/ir.2017.15.1.68 28239315 PMC5323309

[45] Paramsothy S, Kamm MA, Kaakoush NO, Walsh AJ, van den Bogaerde J, et al (2017) Multidonor intensive faecal microbiota transplantation for active ulcerative colitis: a randomised placebo-controlled trial. Lancet 389: 1218–1228. 10.1016/S0140-6736(17)30182-4 28214091

[46] Wei Y, Gong J, Zhu W, Tian H, Ding C, et al (2016) Pectin enhances the effect of fecal microbiota transplantation in ulcerative colitis by delaying the loss of diversity of gut flora. BMC Microbiol 16: 255 10.1186/s12866-016-0869-2 27809778 PMC5095982

[47] Rossen NG, Fuentes S, van der Spek MJ, Tijssen JG, Hartman JH, et al (2015) Findings From a Randomized Controlled Trial of Fecal Transplantation for Patients With Ulcerative Colitis. Gastroenterology 149: 110–118 e114. 10.1053/j.gastro.2015.03.045 25836986

[48] Moayyedi P, Surette MG, Kim PT, Libertucci J, Wolfe M, et al (2015) Fecal Microbiota Transplantation Induces Remission in Patients With Active Ulcerative Colitis in a Randomized Controlled Trial. Gastroenterology 149: 102–109 e106. 10.1053/j.gastro.2015.04.001 25857665

[49] Costello SP, Hughes PA, Waters O, Bryant RV, Vincent AD, et al (2019) Effect of Fecal Microbiota Transplantation on 8-Week Remission in Patients With Ulcerative Colitis: A Randomized Clinical Trial. JAMA 321: 156–164. 10.1001/jama.2018.20046 30644982 PMC6439766

[50] Herfarth H, Barnes EL, Long MD, Isaacs KL, Leith T, et al (2019) Combined Endoscopic and Oral Fecal Microbiota Transplantation in Patients with Antibiotic-Dependent Pouchitis: Low Clinical Efficacy due to Low Donor Microbial Engraftment. Inflamm Intest Dis 4: 1–6. 10.1159/000497042 31172007 PMC6537468

[51] Yang Z, Bu C, Yuan W, Shen Z, Quan Y, et al (2020) Fecal Microbiota Transplant via Endoscopic Delivering Through Small Intestine and Colon: No Difference for Crohn's Disease. Dig Dis Sci 65: 150–157. 10.1007/s10620-019-05751-y 31367877

[52] Sokol H, Landman C, Seksik P, Berard L, Montil M, et al (2020) Fecal microbiota transplantation to maintain remission in Crohn's disease: a pilot randomized controlled study. Microbiome 8: 12 10.1186/s40168-020-0792-5 32014035 PMC6998149

[53] Sood A, Mahajan R, Singh A, Midha V, Mehta V, et al (2019) Role of Faecal Microbiota Transplantation for Maintenance of Remission in Patients With Ulcerative Colitis: A Pilot Study. J Crohns Colitis 13: 1311–1317. 10.1093/ecco-jcc/jjz060 30873549

[54] Lee CH, Steiner T, Petrof EO, Smieja M, Roscoe D, et al (2016) Frozen vs Fresh Fecal Microbiota Transplantation and Clinical Resolution of Diarrhea in Patients With Recurrent Clostridium difficile Infection: A Randomized Clinical Trial. JAMA 315: 142–149. 10.1001/jama.2015.18098 26757463

[55] Satokari R, Mattila E, Kainulainen V, Arkkila PE (2015) Simple faecal preparation and efficacy of frozen inoculum in faecal microbiota transplantation for recurrent Clostridium difficile infection—an observational cohort study. Aliment Pharmacol Ther 41: 46–53. 10.1111/apt.13009 25355279

[56] Hamilton MJ, Weingarden AR, Sadowsky MJ, Khoruts A (2012) Standardized frozen preparation for transplantation of fecal microbiota for recurrent Clostridium difficile infection. Am J Gastroenterol 107: 761–767. 10.1038/ajg.2011.482 22290405

[57] Kim KO, Gluck M (2019) Fecal Microbiota Transplantation: An Update on Clinical Practice. Clin Endosc 52: 137–143. 10.5946/ce.2019.009 30909689 PMC6453848

[58] Papanicolas LE, Choo JM, Wang Y, Leong LEX, Costello SP, et al (2019) Bacterial viability in faecal transplants: Which bacteria survive? EBioMedicine 41: 509–516. 10.1016/j.ebiom.2019.02.023 30796005 PMC6444077

[59] Jung Lee W, Lattimer LD, Stephen S, Borum ML, Doman DB (2015) Fecal Microbiota Transplantation: A Review of Emerging Indications Beyond Relapsing Clostridium difficile Toxin Colitis. Gastroenterol Hepatol (N Y) 11: 24–32. 27099570 PMC4836576

[60] Edelstein C, Daw JR, Kassam Z (2016) Seeking safe stool: Canada needs a universal donor model. CMAJ 188: E431–E432. 10.1503/cmaj.150672 26696614 PMC5135517

[61] Imdad A, Nicholson MR, Tanner-Smith EE, Zackular JP, Gomez-Duarte OG, et al (2018) Fecal transplantation for treatment of inflammatory bowel disease. Cochrane Database Syst Rev 11: CD012774. 10.1002/14651858.CD012774.pub2 PMC6517295 30480772

[62] Paramsothy S, Paramsothy R, Rubin DT, Kamm MA, Kaakoush NO, et al (2017) Faecal Microbiota Transplantation for Inflammatory Bowel Disease: A Systematic Review and Meta-analysis. J Crohns Colitis 11: 1180–1199. 10.1093/ecco-jcc/jjx063 28486648

[63] Qazi T, Amaratunga T, Barnes EL, Fischer M, Kassam Z, et al (2017) The risk of inflammatory bowel disease flares after fecal microbiota transplantation: Systematic review and meta-analysis. Gut Microbes 8: 574–588. 10.1080/19490976.2017.1353848 28723262 PMC5730391

[64] Narula N, Kassam Z, Yuan Y, Colombel JF, Ponsioen C, et al (2017) Systematic Review and Meta-analysis: Fecal Microbiota Transplantation for Treatment of Active Ulcerative Colitis. Inflamm Bowel Dis 23: 1702–1709. 10.1097/MIB.0000000000001228 28906291

[65] Colman RJ, Rubin DT (2014) Fecal microbiota transplantation as therapy for inflammatory bowel disease: a systematic review and meta-analysis. J Crohns Colitis 8: 1569–1581. 10.1016/j.crohns.2014.08.006 25223604 PMC4296742

[66] Anderson JL, Edney RJ, Whelan K (2012) Systematic review: faecal microbiota transplantation in the management of inflammatory bowel disease. Aliment Pharmacol Ther 36: 503–516. 10.1111/j.1365-2036.2012.05220.x 22827693

[67] Konig J, Siebenhaar A, Hogenauer C, Arkkila P, Nieuwdorp M, et al (2017) Consensus report: faecal microbiota transfer—clinical applications and procedures. Aliment Pharmacol Ther 45: 222–239. 10.1111/apt.13868 27891639 PMC6680358

[68] Zhou HY, Guo B, Lufumpa E, Li XM, Chen LH, et al (2020) Comparative of the Effectiveness and Safety of Biological Agents, Tofacitinib, and Fecal Microbiota Transplantation in Ulcerative Colitis: Systematic Review and Network Meta-Analysis. Immunol Invest: 1–15. 10.1080/08820139.2020.1714650 32009472

[69] Dang X, Xu M, Liu D, Zhou D, Yang W (2020) Assessing the efficacy and safety of fecal microbiota transplantation and probiotic VSL#3 for active ulcerative colitis: A systematic review and meta-analysis. PLoS One 15: e0228846 10.1371/journal.pone.0228846 32182248 PMC7077802

[70] Cammarota G, Ianiro G, Kelly CR, Mullish BH, Allegretti JR, et al (2019) International consensus conference on stool banking for faecal microbiota transplantation in clinical practice. Gut 68: 2111–2121. 10.1136/gutjnl-2019-319548 31563878 PMC6872442

[71] Harbord M, Eliakim R, Bettenworth D, Karmiris K, Katsanos K, et al (2017) Third European Evidence-based Consensus on Diagnosis and Management of Ulcerative Colitis. Part 2: Current Management. J Crohns Colitis 11: 769–784. 10.1093/ecco-jcc/jjx009 28513805

[72] Gomollon F, Dignass A, Annese V, Tilg H, Van Assche G, et al (2017) 3rd European Evidence-based Consensus on the Diagnosis and Management of Crohn's Disease 2016: Part 1: Diagnosis and Medical Management. J Crohns Colitis 11: 3–25. 10.1093/ecco-jcc/jjw168 27660341

