# Supplementary material for: Fecal microbiota transplantation in inflammatory bowel disease patients: A systematic review and meta-analysis
Source: PLoS One. 2020 Sep 18;15(9):e0238910. doi: 10.1371/journal.pone.0238910 (PMC7500646; doi:10.1371/journal.pone.0238910)
Supplement: S1 Table — (DOCX) [file pone.0238910.s002.docx]

Table S1. List of the 60 included references.

| 1. | He Z, Cui BT, Zhang T, et al. Fecal microbiota transplantation cured epilepsy in a case with Crohn's disease: The first report. World J Gastroenterol. 2017;23(19):3565-8. |
| --- | --- |
| 2. | Kump P, Wurm P, Grochenig HP, et al. The taxonomic composition of the donor intestinal microbiota is a major factor influencing the efficacy of faecal microbiota transplantation in therapy refractory ulcerative colitis. Aliment Pharmacol Ther. 2018;47(1):67-77. |
| 3. | Goyal A, Yeh A, Bush BR, et al. Safety, Clinical Response, and Microbiome Findings Following Fecal Microbiota Transplant in Children With Inflammatory Bowel Disease. Inflamm Bowel Dis. 2018;24(2):410-21. |
| 4. | Zhu JG, Zhang FM, Zhou JF, et al. Assessment of therapeutic response in Crohn's disease using quantitative dynamic contrast enhanced MRI (DCE-MRI) parameters A preliminary study. Medicine. 2017;96(32). |
| 5. | Uygun A, Ozturk K, Demirci H, et al. Fecal microbiota transplantation is a rescue treatment modality for refractory ulcerative colitis. Medicine (Baltimore). 2017;96(16):e6479. |
| 6. | Paramsothy S, Kamm MA, Kaakoush NO, et al. Multidonor intensive faecal microbiota transplantation for active ulcerative colitis: a randomised placebo-controlled trial. Lancet. 2017;389(10075):1218-28. |
| 7. | Nishida A, Imaeda H, Ohno M, et al. Efficacy and safety of single fecal microbiota transplantation for Japanese patients with mild to moderately active ulcerative colitis. J Gastroenterol. 2017;52(4):476-82. |
| 8. | Mizuno S, Nanki K, Matsuoka K, et al. Single fecal microbiota transplantation failed to change intestinal microbiota and had limited effectiveness against ulcerative colitis in Japanese patients. Intest Res. 2017;15(1):68-74. |
| 9. | Liu SX, Li YH, Dai WK, et al. Fecal microbiota transplantation induces remission of infantile allergic colitis through gut microbiota re-establishment. World J Gastroenterol. 2017;23(48):8570-81. |
| 10. | Karolewska-Bochenek K, Grzesiowski P, Banaszkiewicz A, et al. A Two-Week Fecal Microbiota Transplantation Course in Pediatric Patients with Inflammatory Bowel Disease. Advances in experimental medicine and biology. 2017. |
| 11. | Jacob V, Crawford C, Cohen-Mekelburg S, et al. Single Delivery of High-Diversity Fecal Microbiota Preparation by Colonoscopy Is Safe and Effective in Increasing Microbial Diversity in Active Ulcerative Colitis. Inflamm Bowel Dis. 2017;23(6):903-11. |
| 12. | Ishikawa D, Sasaki T, Osada T, et al. Changes in Intestinal Microbiota Following Combination Therapy with Fecal Microbial Transplantation and Antibiotics for Ulcerative Colitis. Inflamm Bowel Dis. 2017;23(1):116-25. |
| 13. | He Z, Li P, Zhu J, et al. Multiple fresh fecal microbiota transplants induces and maintains clinical remission in Crohn's disease complicated with inflammatory mass. Sci Rep. 2017;7(1):4753. |
| 14. | Gunaltay S, Rademacher L, Hornquist EH, et al. Clinical and immunologic effects of faecal microbiota transplantation in a patient with collagenous colitis. World J Gastroenterol. 2017;23(7):1319-24. |
| 15. | Fang YH, Chen J, Yu JD, et al. The Preliminary Investigation of Faecal Microbiota Transplantation for Paediatric Recurrent Chronic Bowel Diseases and Literature Review. Hong Kong J Paediatr. 2017;22(4):199-203. |
| 16. | Bak SH, Choi HH, Lee J, et al. Fecal microbiota transplantation for refractory Crohn's disease. Intest Res. 2017;15(2):244-8. |
| 17. | Zhang T, Cui BT, Li P, et al. Short-Term Surveillance of Cytokines and C-Reactive Protein Cannot Predict Efficacy of Fecal Microbiota Transplantation for Ulcerative Colitis. PLoS One. 2016;11(6). |
| 18. | Wei Y, Gong JF, Zhu WM, et al. Pectin enhances the effect of fecal microbiota transplantation in ulcerative colitis by delaying the loss of diversity of gut flora. BMC Microbiol. 2016;16. |
| 19. | Vermeire S, Joossens M, Verbeke K, et al. Donor Species Richness Determines Faecal Microbiota Transplantation Success in Inflammatory Bowel Disease. Journal of Crohn's & colitis. 2016;10(4):387-94. |
| 20. | Vaughn BP, Vatanen T, Allegretti JR, et al. Increased Intestinal Microbial Diversity Following Fecal Microbiota Transplant for Active Crohn's Disease. Inflamm Bowel Dis. 2016;22(9):2182-90. |
| 21. | Shimizu H, Arai K, Abe J, et al. Repeated fecal microbiota transplantation in a child with ulcerative colitis. Pediatr Int. 2016;58(8):781-5. |
| 22. | Seth AK, Rawal P, Bagga R, et al. Successful colonoscopic fecal microbiota transplantation for active ulcerative colitis: First report from India. Indian J Gastroenterol. 2016;35(5):393-5. |
| 23. | Oprita R, Bratu M, Oprita B, et al. Fecal transplantation - the new, inexpensive, safe, and rapidly effective approach in the treatment of gastrointestinal tract diseases. Journal of medicine and life. 2016;9(2):160-2. |
| 24. | Ni XD, Fan SX, Zhang YL, et al. Coordinated Hospital-Home Fecal Microbiota Transplantation via Percutaneous Endoscopic Cecostomy for Recurrent Steroid-Dependent Ulcerative Colitis. Gut Liver. 2016;10(6):975-80. |
| 25. | Laszlo M, Ciobanu L, Andreica V, et al. Fecal transplantation indications in ulcerative colitis. Preliminary study. Clujul medical (1957). 2016;89(2):224-8. |
| 26. | Kumagai H, Yokoyama K, Imagawa T, et al. Failure of Fecal Microbiota Transplantation in a Three-Year-Old Child with Severe Refractory Ulcerative Colitis. Pediatr Gastroenterol Hepatol Nutr. 2016;19(3):214-20. |
| 27. | Wei Y, Zhu W, Gong J, et al. Fecal Microbiota Transplantation Improves the Quality of Life in Patients with Inflammatory Bowel Disease. Gastroenterol Res Pract. 2015;2015:517597. |
| 28. | Vandenplas Y, Veereman G, van der Werff Ten Bosch J, et al. Fecal Microbial Transplantation in Early-Onset Colitis: Caution Advised. J Pediatr Gastroenterol Nutr. 2015;61(3):e12-4. |
| 29. | Rossen NG, Fuentes S, van der Spek MJ, et al. Findings From a Randomized Controlled Trial of Fecal Transplantation for Patients With Ulcerative Colitis. Gastroenterology. 2015;149(1):110. |
| 30. | Moayyedi P, Surette MG, Kim PT, et al. Fecal Microbiota Transplantation Induces Remission in Patients With Active Ulcerative Colitis in a Randomized Controlled Trial. Gastroenterology. 2015;149(1):102. |
| 31. | Damman CJ, Brittnacher MJ, Westerhoff M, et al. Low Level Engraftment and Improvement following a Single Colonoscopic Administration of Fecal Microbiota to Patients with Ulcerative Colitis. PLoS One. 2015;10(8). |
| 32. | Cui BT, Li P, Xu LJ, et al. Step-up fecal microbiota transplantation strategy: a pilot study for steroid-dependent ulcerative colitis. J Transl Med. 2015;13. |
| 33. | Cui BT, Feng Q, Wang HG, et al. Fecal microbiota transplantation through mid-gut for refractory Crohn's disease: Safety, feasibility, and efficacy trial results. J Gastroenterol Hepatol. 2015;30(1):51-8. |
| 34. | Kao DN, Hotte N, Gillevet P, et al. Fecal Microbiota Transplantation Inducing Remission in Crohn's Colitis and the Associated Changes in Fecal Microbial Profile. J Clin Gastroenterol. 2014;48(7):625-8. |
| 35. | Zhang FM, Wang HG, Wang M, et al. Fecal microbiota transplantation for severe enterocolonic fistulizing Crohn's disease. World J Gastroenterol. 2013;19(41):7213-6. |
| 36. | Kunde S, Pham A, Bonczyk S, et al. Safety, Tolerability, and Clinical Response After Fecal Transplantation in Children and Young Adults With Ulcerative Colitis. J Pediatr Gastroenterol Nutr. 2013;56(6):597-601. |
| 37. | Kump PK, Grochenig HP, Lackner S, et al. Alteration of Intestinal Dysbiosis by Fecal Microbiota Transplantation Does not Induce Remission in Patients with Chronic Active Ulcerative Colitis. Inflamm Bowel Dis. 2013;19(10):2155-65. |
| 38. | Angelberger S, Reinisch W, Makristathis A, et al. Temporal Bacterial Community Dynamics Vary Among Ulcerative Colitis Patients After Fecal Microbiota Transplantation. Am J Gastroenterol. 2013;108(10):1620-30. |
| 39. | Borody TJ, Warren EF, Leis S, et al. Treatment of ulcerative colitis using fecal bacteriotherapy. J Clin Gastroenterol. 2003;37(1):42-7. |
| 40. | Landy J, Walker AW, Li JV, et al. Variable alterations of the microbiota, without metabolic or immunological change, following faecal microbiota transplantation in patients with chronic pouchitis. Sci Rep. 2015;5:12955. |
| 41. | Suskind DL, Brittnacher MJ, Wahbeh G, et al. Fecal microbial transplant effect on clinical outcomes and fecal microbiome in active Crohn's disease. Inflammatory bowel diseases. 2015;21(3):556-63. |
| 42. | Suskind DL, Singh N, Nielson H, et al. Fecal microbial transplant via nasogastric tube for active pediatric ulcerative colitis. Journal of pediatric gastroenterology and nutrition. 2015;60(1):27-9. |
| 43. | Costello SP, Hughes PA, Waters O, et al. Effect of Fecal Microbiota Transplantation on 8-Week Remission in Patients With Ulcerative Colitis: A Randomized Clinical Trial. Jama. 2019;321(2):156-64. |
| 44. | Sood A, Mahajan R, Juyal G, et al. Efficacy of fecal microbiota therapy in steroid dependent ulcerative colitis: a real world intention-to-treat analysis. Intestinal research. 2019;17(1):78-86. |
| 45. | Wang H, Cui B, Li Q, et al. The Safety of Fecal Microbiota Transplantation for Crohn's Disease: Findings from A Long-Term Study. Advances in therapy. 2018;35(11):1935-44. |
| 46. | Sood A, Singh A, Mahajan R, et al. Acceptability, tolerability, and safety of fecal microbiota transplantation in patients with active ulcerative colitis (AT&S Study). J Gastroenterol Hepatol. 2020;35(3):418-24. |
| 47. | Adler E, Tabaa A, Kassam Z, et al. Capsule-Delivered Fecal Microbiota Transplant Is Safe and Well Tolerated in Patients with Ulcerative Colitis. Dig Dis Sci. 2019;64(9):2452-4 |
| 48. | Herfarth H, Barnes EL, Long MD, et al. Combined Endoscopic and Oral Fecal Microbiota Transplantation in Patients with Antibiotic-Dependent Pouchitis: Low Clinical Efficacy due to Low Donor Microbial Engraftment. Inflammatory intestinal diseases. 2019;4(1):1-6. |
| 49. | Xiang L, Ding X, Li Q, et al. Efficacy of faecal microbiota transplantation in Crohn's disease: a new target treatment? Microb Biotechnol. 2020. |
| 50. | Nishida A, Imaeda H, Inatomi O, et al. The efficacy of fecal microbiota transplantation for patients with chronic pouchitis: A case series. Clin Case Rep. 2019;7(4):782-8. |
| 51. | Tariq R, Disbrow MB, Dibaise JK, et al. Efficacy of Fecal Microbiota Transplantation for Recurrent C. Difficile Infection in Inflammatory Bowel Disease. Inflamm Bowel Dis. 2019. |
| 52. | Gutin L, Piceno Y, Fadrosh D, et al. Fecal microbiota transplant for Crohn disease: A study evaluating safety, efficacy, and microbiome profile. United European Gastroenterol J. 2019;7(6):807-14. |
| 53. | Yang Z, Bu C, Yuan W, et al. Fecal Microbiota Transplant via Endoscopic Delivering Through Small Intestine and Colon: No Difference for Crohn's Disease. Dig Dis Sci. 2020;65(1):150-7. |
| 54. | Tian Y, Zhou Y, Huang S, et al. Fecal microbiota transplantation for ulcerative colitis: a prospective clinical study. BMC Gastroenterol. 2019;19(1):116. |
| 55. | Selvig D, Piceno Y, Terdiman J, et al. Fecal Microbiota Transplantation in Pouchitis: Clinical, Endoscopic, Histologic, and Microbiota Results from a Pilot Study. Dig Dis Sci. 2020;65(4):1099-106. |
| 56. | Zou M, Jie Z, Cui B, et al. Fecal microbiota transplantation results in bacterial strain displacement in patients with inflammatory bowel diseases. FEBS Open Bio. 2020;10(1):41-55. |
| 57. | Sokol H, Landman C, Seksik P, et al. Fecal microbiota transplantation to maintain remission in Crohn's disease: a pilot randomized controlled study. Microbiome. 2020;8(1):12. |
| 58. | Wang JW, Wang YK, Zhang F, et al. Initial experience of fecal microbiota transplantation in gastrointestinal disease: A case series. The Kaohsiung journal of medical sciences. 2019;35(9):566-71. |
| 59. | Cold F, Browne PD, Gunther S, et al. Multidonor FMT capsules improve symptoms and decrease fecal calprotectin in ulcerative colitis patients while treated - an open-label pilot study. Scand J Gastroenterol. 2019;54(3):289-96. |
| 60. | Sood A, Mahajan R, Singh A, et al. Role of Faecal Microbiota Transplantation for Maintenance of Remission in Patients With Ulcerative Colitis: A Pilot Study. Journal of Crohn's & colitis. 2019;13(10):1311-7. |
