# Supplementary figures and images for: Fecal microbiota transplantation in inflammatory bowel disease patients: A systematic review and meta-analysis
Source: PLoS One. 2020 Sep 18;15(9):e0238910. doi: 10.1371/journal.pone.0238910 (PMC7500646; doi:10.1371/journal.pone.0238910)

Figure S1. Risk of bias graph of included RCTs.


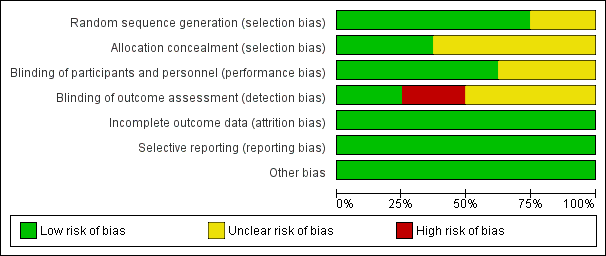

Supplement: S1 Fig — (DOCX) [file pone.0238910.s004.docx]

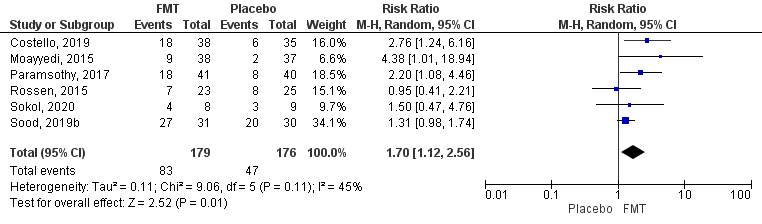
Figure S2. Forest plot for clinical remission.

Supplement: S2 Fig — (DOCX) [file pone.0238910.s005.docx]

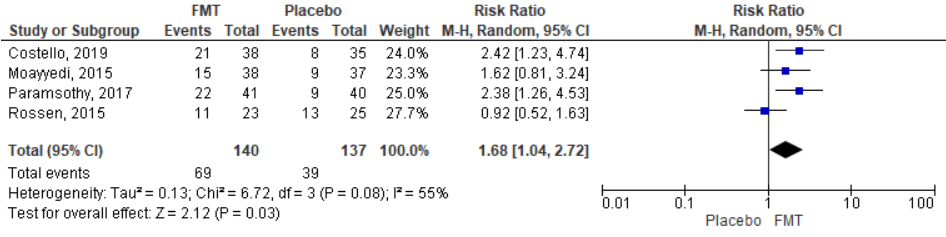
Figure S3. Forest plot for clinical response.

Supplement: S3 Fig — (DOCX) [file pone.0238910.s006.docx]

Figure S4. Clinical remission - subgroup analysis by stool type.


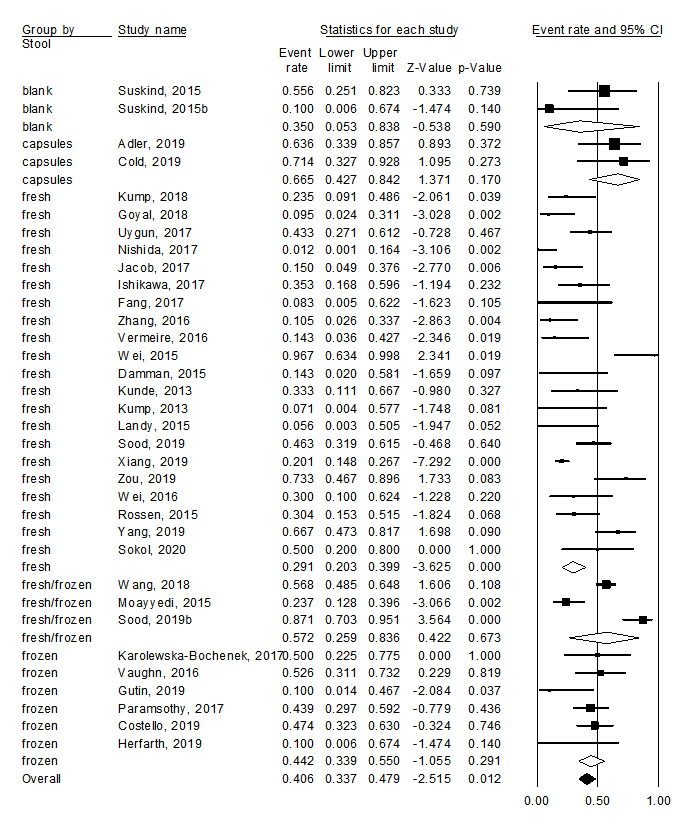

Supplement: S4 Fig — (DOCX) [file pone.0238910.s007.docx]

Figure S5. Clinical remission - subgroup analysis by donor type.


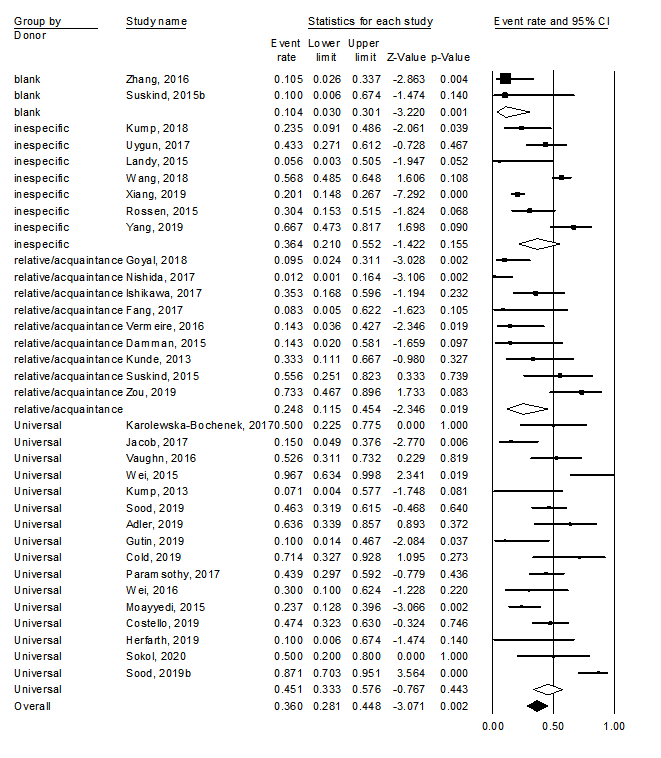

Supplement: S5 Fig — (DOCX) [file pone.0238910.s008.docx]

Figure S6. Clinical remission - subgroup analysis by IBD subtype.


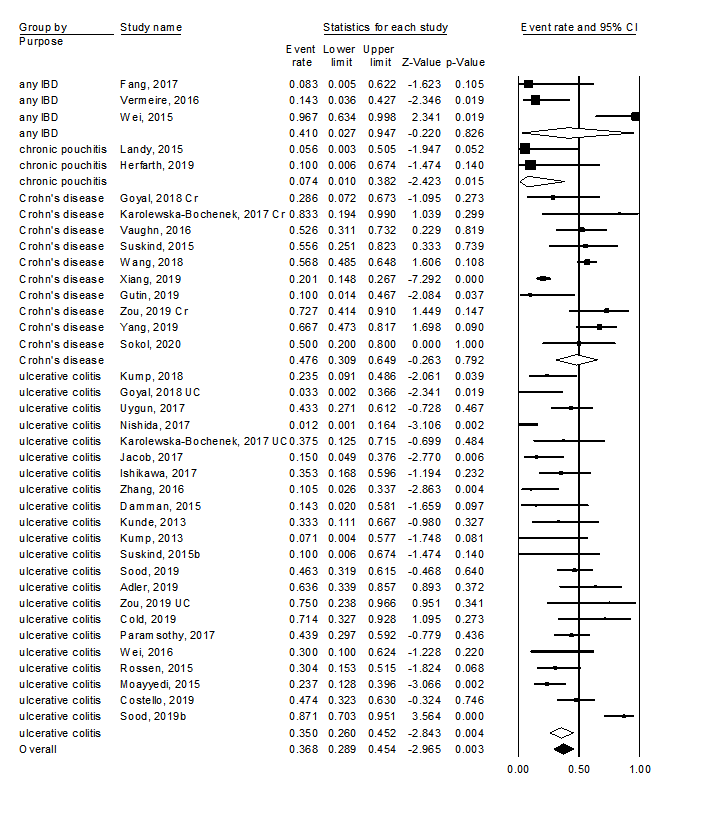

Supplement: S6 Fig — (DOCX) [file pone.0238910.s009.docx]

Figure S7. Sensitivity analysis for clinical remission.


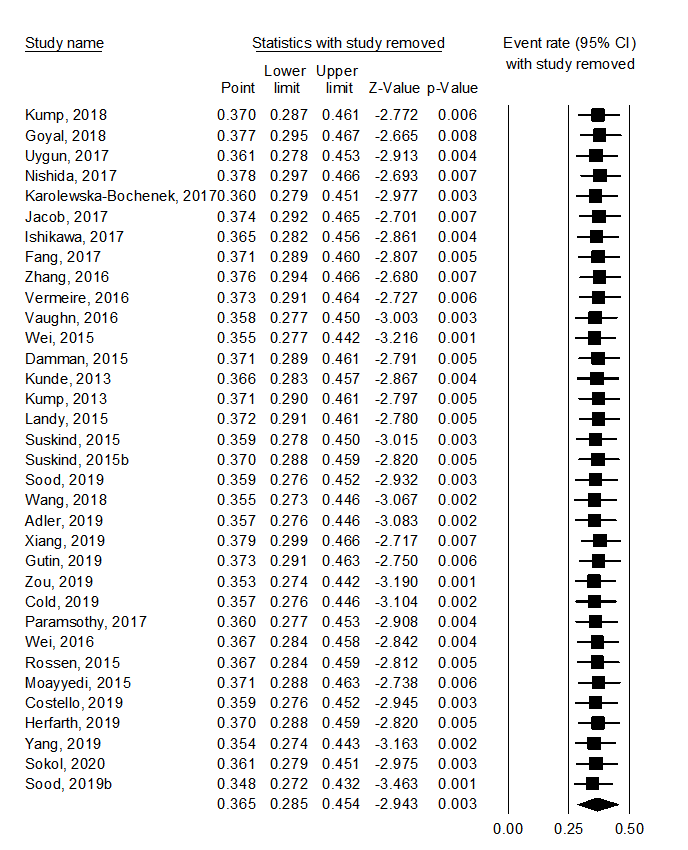

Supplement: S7 Fig — (DOCX) [file pone.0238910.s010.docx]

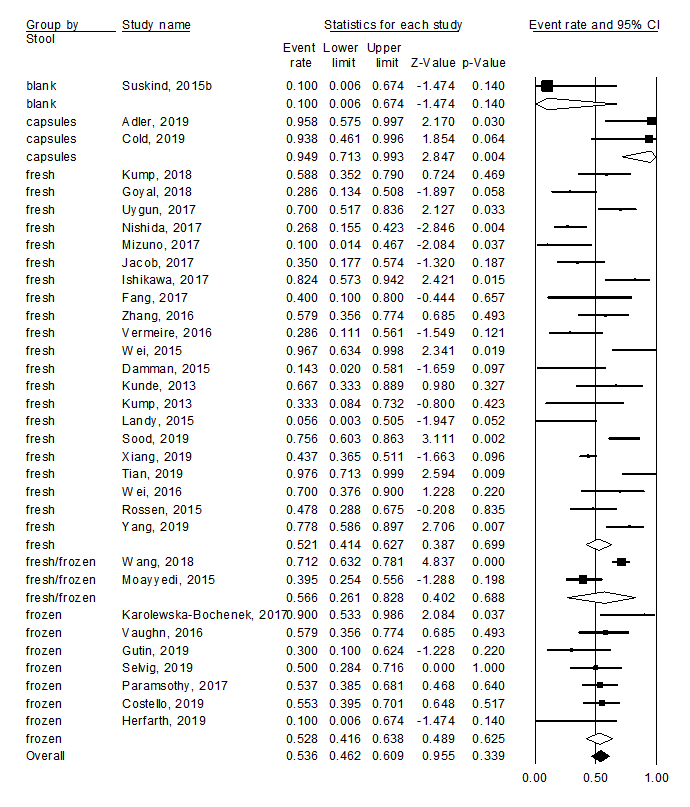
Figure S8. Clinical response - subgroup analysis by stool type.

Supplement: S8 Fig — (DOCX) [file pone.0238910.s011.docx]

Figure S9. Clinical response - subgroup analysis by donor type.


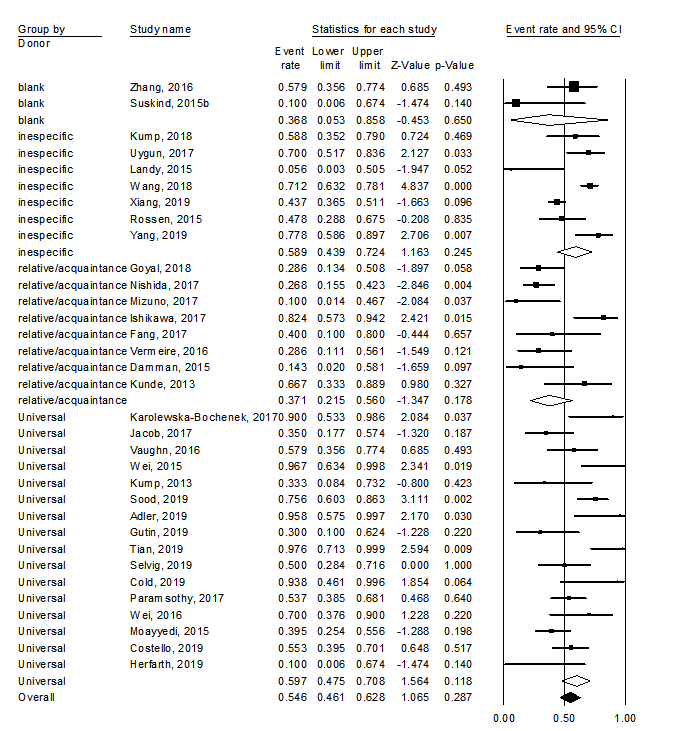

Supplement: S9 Fig — (DOCX) [file pone.0238910.s012.docx]

Figure S10. Clinical response - subgroup analysis by IBD subtype.


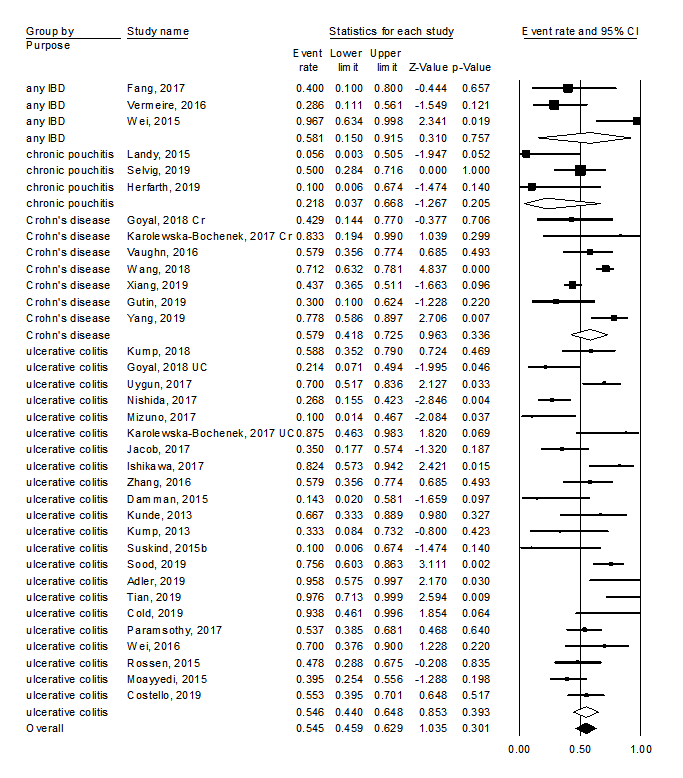

Supplement: S10 Fig — (DOCX) [file pone.0238910.s013.docx]

Figure S11. Sensitivity analysis for clinical response.


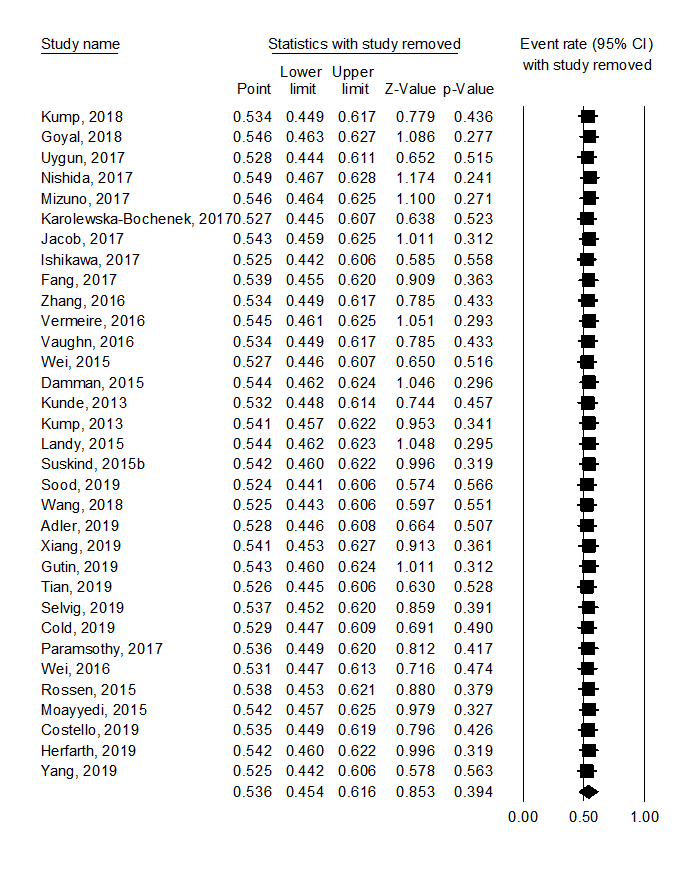

Supplement: S11 Fig — (DOCX) [file pone.0238910.s014.docx]

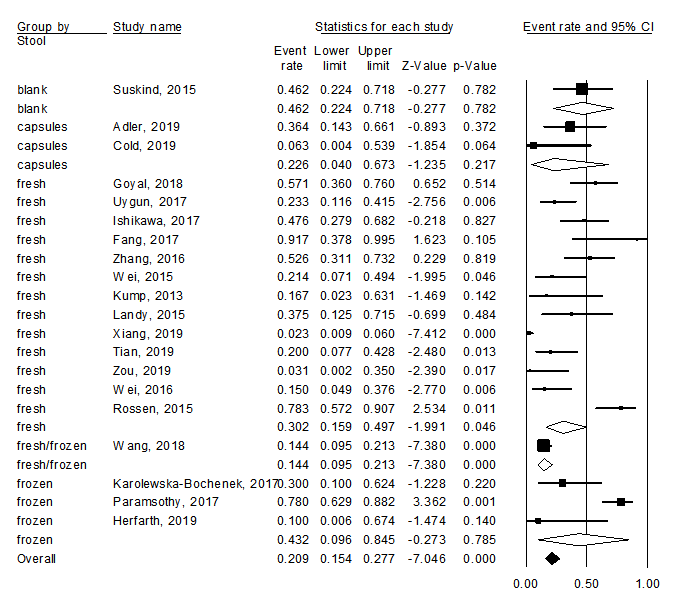
Figure S12. Any adverse event - subgroup analysis by stool type.

Supplement: S12 Fig — (DOCX) [file pone.0238910.s015.docx]

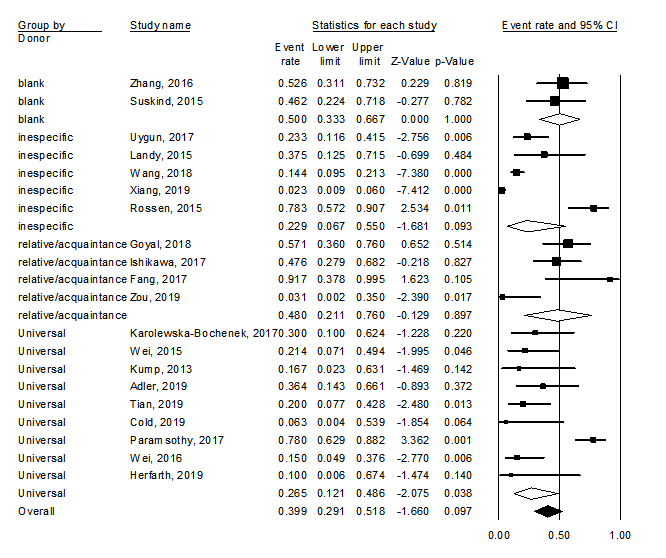
Figure S13. Any adverse event - subgroup analysis by donor type.

Supplement: S13 Fig — (DOCX) [file pone.0238910.s016.docx]

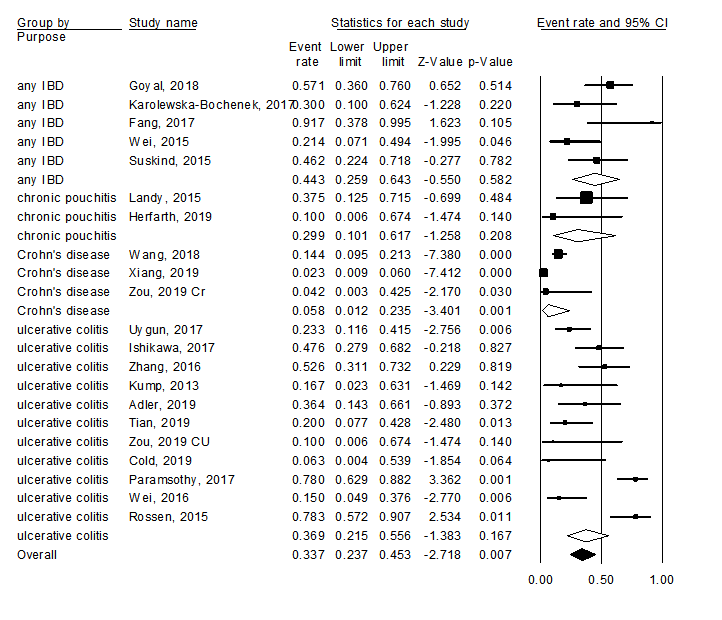
Figure S14. Any adverse event - subgroup analysis by IBD subtype.

Supplement: S14 Fig — (DOCX) [file pone.0238910.s017.docx]

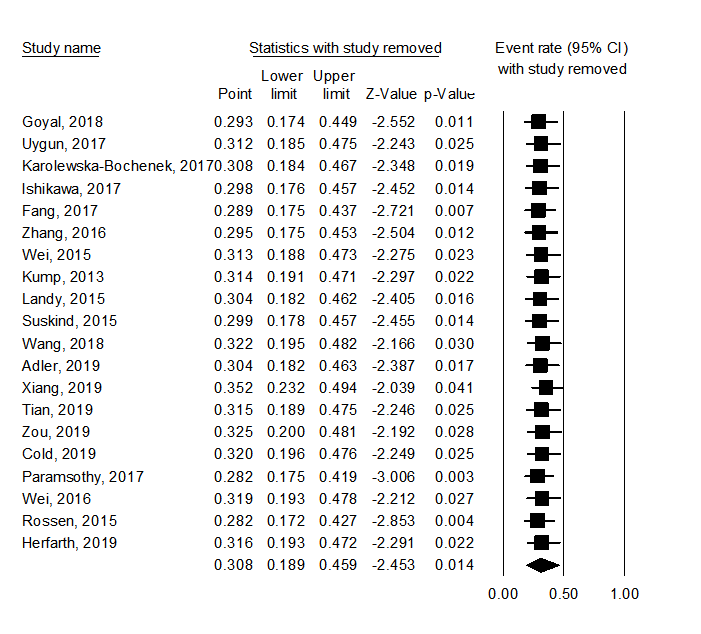
Figure S15. Sensitivity analysis for any adverse event.

Supplement: S15 Fig — (DOCX) [file pone.0238910.s018.docx]

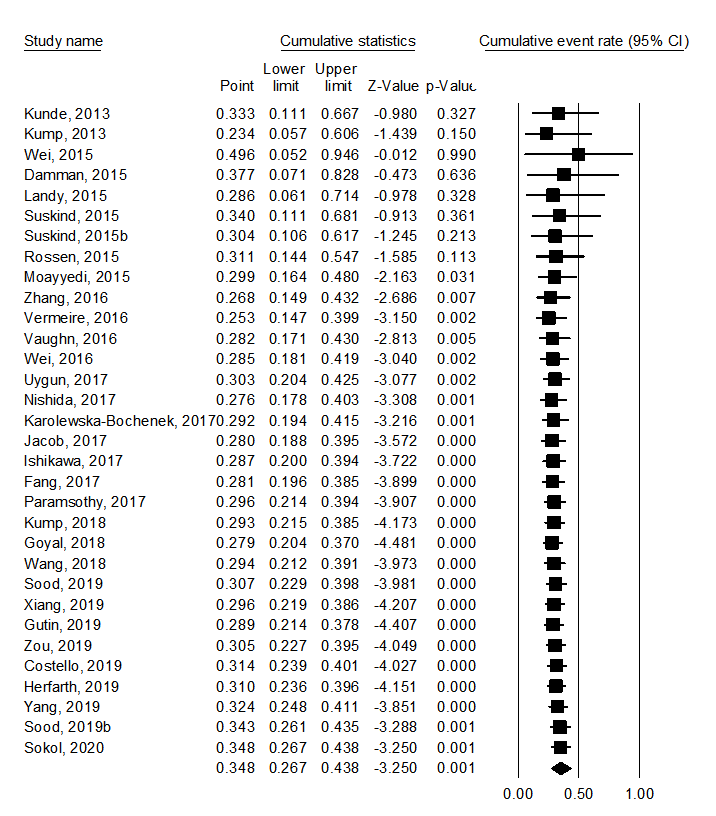
Figure S16. Cumulative meta-analysis for clinical remission.

Supplement: S16 Fig — (DOCX) [file pone.0238910.s019.docx]

Figure S17. Funnel plot for assessment of potential publication bias.


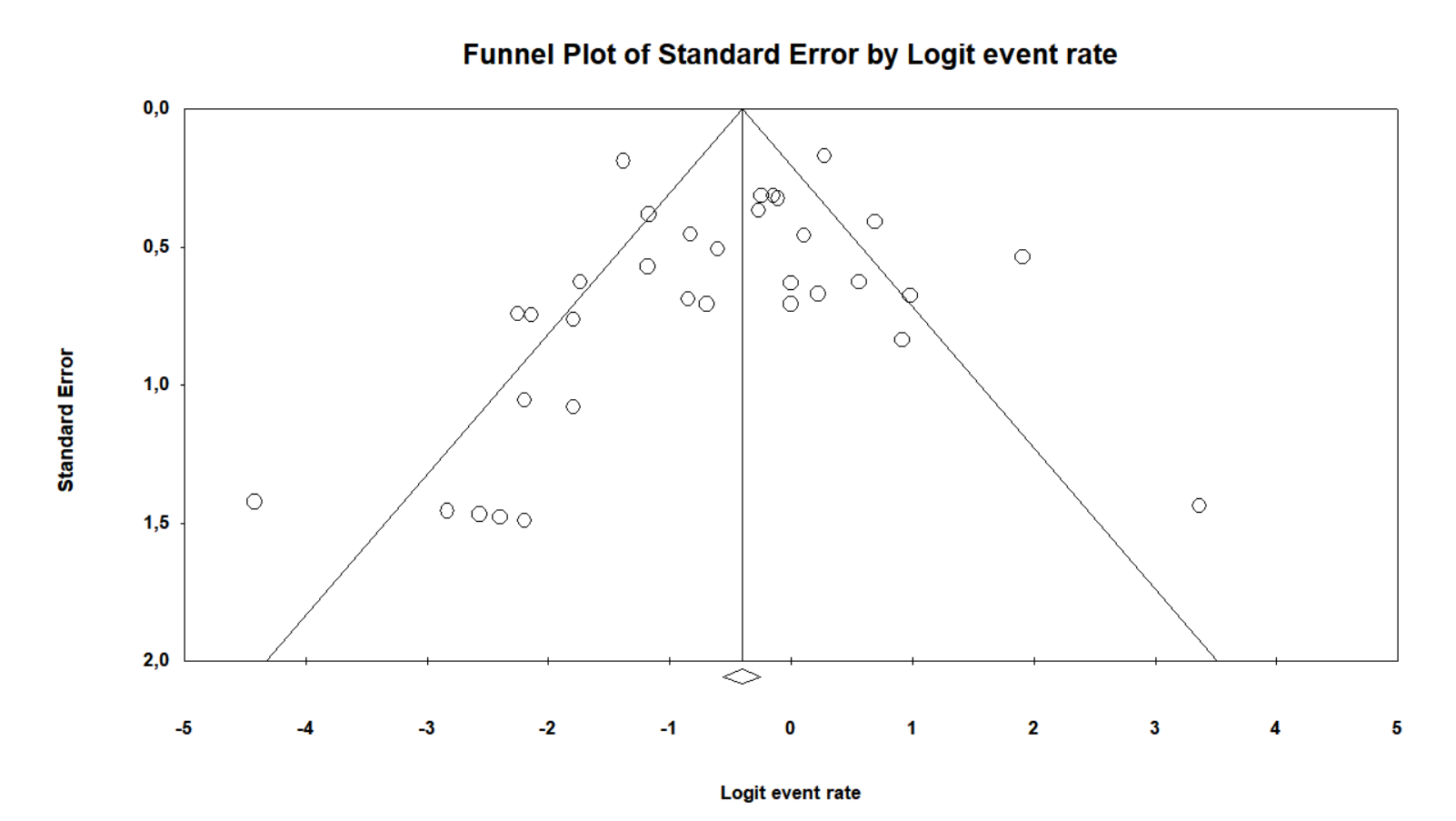

Supplement: S17 Fig — (DOCX) [file pone.0238910.s020.docx]
